# Immunotherapy targeting pyroglutamate-3 Aβ: prospects and challenges

**DOI:** 10.1186/s13024-016-0115-2

**Published:** 2016-06-30

**Authors:** Holger Cynis, Jeffrey L. Frost, Helen Crehan, Cynthia A. Lemere

**Affiliations:** Ann Romney Center for Neurologic Diseases, Brigham and Women’s Hospital, Harvard Medical School, 77 Avenue Louis Pasteur, NRB636, Boston, MA 02115 USA; Fraunhofer Institute for Cell Therapy and Immunology, Weinbergweg 22, 06120 Halle, Germany; University of Massachusetts Medical School, 55 Lake Avenue North, Worcester, MA 01605 USA

**Keywords:** Amyloid-β, Pyroglutamate-3 Aβ, Immunotherapy, Vaccine, Glutaminyl cyclases

## Abstract

Immunization against amyloid-β (Aβ) peptides deposited in Alzheimer’s disease (AD) has shown considerable therapeutic effect in animal models however, the translation into human Alzheimer’s patients is challenging. In recent years, a number of promising Aβ immunotherapy trials failed to reach primary study endpoints. Aside from uncertainties in the selection of patients and the start and duration of treatment, these results also suggest that the mechanisms underlying AD are still not fully understood. Thorough characterizations of protein aggregates in AD brain have revealed a conspicuous heterogeneity of Aβ peptides enabling the study of the toxic potential of each of the major forms. One such form, amino-terminally truncated and modified pyroglutamate (pGlu)-3 Aβ peptide appears to play a seminal role for disease initiation, qualifying it as novel target for immunotherapy approaches.

## Background

Alzheimer’s disease (AD) is the leading cause of dementia estimated to affect ~80–131.5 million individuals worldwide by the middle of the 21^st^ century [1, 2]. AD is characterized by a progressive loss of memory accompanied by emotional changes, hallucinations, delusions and impaired social behavior leading to an increased need for around-the-clock care in the final stages of the disease [1, 3]. The disorder is considered to be developed spontaneously in the vast majority of individuals, although studies in monozygous twins suggest a strong genetic component [4]. The average age of onset is within the 7^th^ or 8^th^ decade of life. AD is characterized by two pathological hallmarks, extracellular amyloid plaques composed of deposited β-amyloid (Aβ) peptides, and intraneuronal neurofibrillary tangles formed from hyperphosphorylated tau protein [4]. In a low number of AD cases (<1 %) the disease is inherited in a dominant fashion. Most of the identified familial AD (FAD) mutations lie within the sequence of the amyloid precursor protein (APP) or in the presenilins, the proteolytic machinery that liberates Aβ molecules [3, 5]. The resulting alterations in APP processing or changes in the biophysical properties of the resulting Aβ peptides, lead to an early onset of the disease most frequently developed within the 5^th^ and 6^th^ decade of life [6, 7]; however, some rare mutations are able to cause a shift of the age of onset as early as the 3^rd^ decade of life [8]. Therefore, the accumulation of Aβ aggregates plays an early and essential role in AD and is the target for immunotherapy approaches. However, most Aβ immunization trials have failed thus far. We suggest that one of the possible contributing factors for study failure might be the Aβ forms being targeted by the vaccines and/or antibodies. Previous studies have revealed a heterogeneous mixture of different Aβ species in human brain parenchyma with differing amyloidogenic potential. An N-terminally truncated and pyroglutamate-modified Aβ variant, pGlu-3 Aβ, has gained attention due to its exceptionally high amyloidogeneity and neurotoxicity. Here, we discuss this variant and its potential as a target for Aβ immunization in the light of recent developments in several general Aβ immunotherapy trials.

## Main Text

### The origin and pathobiology of pGlu-3 Aβ

**Fig. 1:**
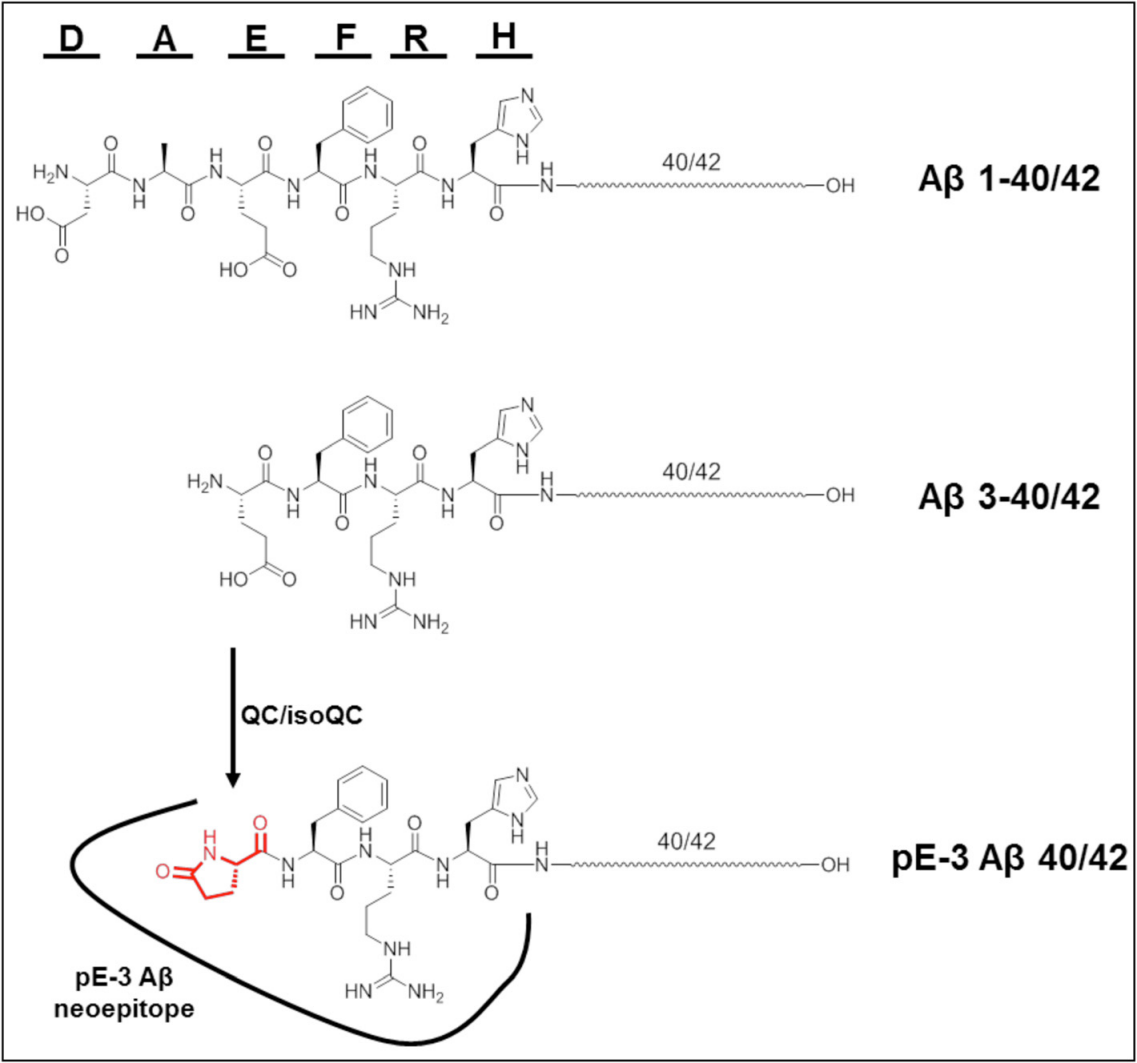
Targeting site for pGlu-3 Aβ-specific therapeutic antibodies. Full-length Aβ is comprised of 40 or 42 amino acids (Aβ 1-40/42). The six N-terminal amino acids of Aβ are depicted by one-letter code of amino acids and chemical structure. pGlu-3 Aβ is a truncated and post-translationally modified variant generated by catalysis of yet unknown proteases and QC/isoQC to convert an N-terminal glutamate residue into a cyclic 5-oxo-proline ring structure (“pGlu”, “pE”). Thereby, a neoepitope is generated that is not present in full-length Aβ molecules. The interaction site of pGlu-3 Aβ-specific antibodies is represented by the line drawn around the pGlu-modified Aβ N-terminus

One major pathologic hallmark of AD is the extracellular amyloid plaque. Amyloid plaque deposits were among the fundamental findings when Alois Alzheimer first described the neurologic disorder later named after him. For a long time the nature of the plaque was unknown until it was found that a protein fragment called amyloid-β (Aβ), generated by cleavage of the large transmembrane protein, amyloid precursor protein (APP), was its main component [9]. The generation of Aβ peptides is orchestrated by the consecutive cleavage of APP by proteases termed β- and γ-secretase. Later, BACE I was identified to be the major β-secretase while a protein complex composed of presenilin 1 (PS1), nicastrin, APH-1 and PEN-2 was identified as γ-secretase [10, 11]. The major Aβ isoforms possess a length of 40 (Aβ40) and 42 (Aβ42) amino acids, respectively. Following β-secretase cleavage of the Aβ N-terminus, the C-terminal isoforms are generated by intramembrane proteolysis by the γ-secretase complex [12]. The major N-terminal form of Aβ was shown to start with an aspartic acid (amino acid 597 of the 695 amino acids long APP isoform) (Fig. 1) [13]. This is in line with the cleavage pattern of BACE I, preferentially leading to the liberation of Aβ peptides starting at Asp-1 [14, 15]. However, reports published shortly after the seminal work by Glenner and Wong [16] identifying Aβ protein, noted truncated and blocked N-termini on Aβ molecules isolated from AD brain [17, 18]. Later it became evident that N-terminal truncated and pGlu-modified Aβ species (pGlu-3 Aβ) in particular are present in large quantities in human AD brain, which is in contrast to the vast majority of AD mouse models, where full-length peptides are the predominant Aβ species [19, 20]. Depending on the method of detection, the relative amount of pGlu-3 Aβ in human brain was described between 1 % as measured by ELISA [21] and 27 % as measured by mass spectrometry [22]. This was recently corroborated by detailed analysis of regional and temporal appearance of general Aβ and pGlu-3 Aβ in sporadic AD, animal models of spontaneous cerebral amyloidosis and transgenic mouse models. In the human AD brain, pGlu-3 Aβ is a major species in diffuse and compacted plaques as well as cerebral amyloid angiopathy, whereas in common transgenic mouse models, pGlu-3 Aβ is deposited only in a subset of compacted plaques and vascular amyloid in later stages of cerebral amyloidosis [23]. Since mouse models have been extensively used in AD research thus far, the low frequency of pGlu-3 Aβ in such models may have hampered investigations on the contribution of these peptides to amyloid pathology.

The production of truncated pGlu-3 Aβ peptides is not fully understood. The identified β-secretase, BACE 1, preferentially generates full-length Aβ peptides starting at Asp-1 and to a lesser extent N-terminally truncated Glu-11 Aβ [11, 14]. However, the generation of pGlu-3 Aβ from Asp-1 Aβ requires 2 additional modifying steps, N-terminal proteolysis of the first two Aβ amino acids followed by cyclization of glutamate at position 3. The mechanism of glutamate cyclization has recently been uncovered and Glutaminyl Cyclase (QC), an aminoacyltransferase was identified as the responsible enzyme *in vitro* [24] and *in vivo* [21] (Fig. 1). This was unexpected, as QC preferentially converts N-terminal glutamine residues into pGlu, whereas the precursor in Aβ is glutamate. However, the substrate specificity of QC is slightly relaxed and QC is also capable of converting, e.g. N-terminal β-homoglutamine [25] finally leading to the discovery of glutamate cyclization by QC under mildly acidic conditions [24]. QC converts glutamate residues at a 3 orders-of-magnitude lower rate constant than N-terminal glutamine residues, but the rate enhancement of QC-mediated catalysis compared to spontaneous cyclization in water is only 2 orders-of-magnitude lower for N-terminal glutamate compared to glutamine. Therefore, the specificity for its primary substrate glutamine is only modest and in turn, QCs catalytic contribution to the generation of pathologic Aβ peptides formed from a glutamate precursor can be considered as substantial [26].

In contrast, the mechanism of N-terminal truncation remains elusive. It has been suggested that Aminopeptidase A contributes to the N-terminal truncation of full-length Aβ peptides [27] but direct liberation of truncated Aβ peptides by alternative pathways have also been suggested [28–31].

Compared to full-length Aβ1-40 and Aβ1-42, pGlu-3 Aβ possesses a higher amyloidogeneity and toxicity. This is reflected by a higher surface hydrophobicity, lower solubility and a strikingly different appearance of fibrils by electron microscopy compared to Aβ1-40 or Aβ1-42 [32, 33]. Most interestingly, pGlu-3 Aβ has been suggested to act as a seed for template-induced misfolding, reminiscent of prions [34]. Co-oligomerization of a low amount of pGlu-3 Aβ peptides with an excess of full-length Aβ1-42 gave rise to the same cytotoxic Aβ species as oligomerization of pGlu-3 Aβ alone. These results suggest that pGlu-3 Aβ oligomers transfer their cytotoxic nature to Aβ1-42 peptides even when present in small quantities and therefore propagate the generation of cytotoxic oligomers in a prion-like fashion [34]. As a consequence, pGlu-3 Aβ molecules might play an important role for early neuronal toxicity observed in AD. Indeed, pGlu-3 Aβ peptides are more toxic to neuronal and glial cultures than full-length Aβ peptides [35]. In addition, overexpression of pGlu-3 Aβ in mice leads to neuron loss [36, 37], which is in stark contrast to the overexpression of Aβ1-40 or 1-42. Both full-length peptides failed to produce an overt neurotoxic phenotype aside from plaque deposition in mice [38, 39]. However, the molecular basis for the higher toxicity of pGlu-3 Aβ is only poorly understood. A potential mechanism suggests that larger pGlu-3 Aβ oligomers are formed through faster aggregation kinetics (compared to full-length Aβ peptides) and subsequently insert into membranes, forming pore-like structures. This might lead to higher ion permeability and altered membrane properties, which ultimately could result in a loss of cell homeostasis [40, 41]. In addition, pGlu-3 Aβ was only recently described to enhance lipid peroxidation in primary cortical mouse neurons, to trigger Ca^2+^ influx and to facilitate the loss of plasma membrane integrity [42].

Consequently, pGlu-3 Aβ is an emerging target for immunotherapy approaches in AD, which have previously shown considerable effects in preclinical animal models [43]. In this regard, the relatively novel concept of anti-pGlu-3 Aβ immunization may benefit from a decade of experiences with immunotherapy trials against other Aβ forms.

### Immunotherapy against Aβ

Immunotherapy against Aβ peptides in AD includes both active and passive immunization approaches [44]. The goal of active Aβ vaccines is to elicit a strong cellular and humoral antibody response through B- and T-cells. This is commonly achieved by using an antigen, either alone or conjugated to a non-self T helper cell epitope, to direct the immune response towards production of anti-Aβ antibodies, combined with an adjuvant to induce high antibody titers. One main advantage of this approach is a sustained immune response that is refined through affinity maturation, thus requiring a limited number of immunizations and making it ideal for distribution in a large patient population. However, caveats are the possibility of an induction of a deleterious inflammatory T-cell response that requires time to quench. In addition, the production of polyclonal antibodies makes this strategy less preferable if specific targeting of a particular isoform or epitope of Aβ is desired. Finally, age-related immunosenescence in the elderly render AD patients poor antibody responders.

Passive immunotherapeutic approaches against Aβ can be utilized to overcome some of these limitations. With direct injection of monoclonal antibodies, immunosenescence is not an issue and specific targeting of a particular epitope or isoform of Aβ is easily achieved. Furthermore, if adverse events should ensue, monoclonal antibody infusions can be discontinued. Drawbacks to this method include sustained production costs associated with generating massive amounts of humanized monoclonal antibodies, continued antibody infusions requiring many visits to the doctor’s office, and the potential of a patient developing anti-antibodies causing a neutralizing effect towards the exogenous antibody injections.

### Recent active Aβ vaccine trials

The promising idea of active vaccination against Aβ peptides came to a sudden halt in 2002, when the first active vaccination trial in humans (AN1792) caused abnormalities described as a form of meningoencephalitis, likely an autoimmune reaction, possibly to Aβ in blood vessel walls, in approximately 6 % of enrolled moderate-to-severe AD patients [45, 46]. The side effects included the presence of lymphocytes in the CSF and focal white matter abnormalities on imaging [45]. While the exact causes of these adverse events were never definitively understood, probable causes may have been the use of full-length Aβ1-42 as an the immunogen, which has since been shown to display several T-cell epitopes particularly in the region between amino acids 15 and 42, the use of Q21, a highly Th1 biased adjuvant, or the formulation change of adding polysorbate 80 for stability [47–49]. Besides the observed adverse events, autopsies of cases enrolled in AN1792 showed evidence of amyloid removal, although without any improvement in dementia [50]. Therefore, second-generation active Aβ vaccines are under development to overcome the pitfalls observed in the AN1792 trial. Active vaccines brought to early stage clinical trials aim to exclusively target B-cell epitopes to generate a robust humoral response, while reducing the chance of an autoimmune T-cell pro-inflammatory response through the use of N-terminal Aβ fragments, mimotope and neoepitope vaccines, as well as phage and virus-like particle vaccines [44].

In 2013, Pfizer and Janssen completed phase II clinical trials in 360 mild to moderate AD patients testing the safety and efficacy of their ACC-001 (vanutide cridificar) active vaccine (https://clinicaltrials.gov), which consists of an Aβ N-terminal fragment (Aβ 1-7) conjugated to an immuno-stimulatory carrier protein with and without QS21 as adjuvant [51]. Recently, the safety outcome of a study in Japanese subjects with mild to moderate AD was published [52]. High antibody titers were reported along with a number of mild to severe adverse events [52]. The clinical development of this treatment was discontinued, in 2015, for reasons that have not been disclosed.

Furthermore, in 2012, Novartis reported its phase I clinical data in patients with mild to moderate AD immunized with CAD106, a novel vaccine consisting of multiple copies of Aβ 1-6 coupled to a coat protein of bacteriophage Qβ. After three subcutaneous injections of 50 μg CAD106 in cohort 1 or 150 μg CAD106 in cohort 2, 67 and 82 %, respectively, of treated patients had generated significant anti-Aβ serum antibodies levels classifying them as responders. While no alterations in CSF Aβ or whole brain MRI volume were observed between CAD106 and placebo groups, these studies were not statistically powered to detect changes in biomarkers [53]. Subsequent phase II clinical trials with CAD106 have been completed and were first reported at the 2014 Alzheimer’s Association International Conference (AAIC) in Copenhagen, Denmark. There, it was reported, that strong serological responders bound less florbetapir at 78 weeks than at baseline. No changes in CSF Aβ were observed, but CSF p-tau declined in strong antibody responders (www.alzforum.org). Additional phase II clinical data were reported only recently [54]. Immunization of patients using 150 μg CAD106 led to 63.8 % serological responders. In addition, 74.5 % adverse events in treated patients compared to 63.3 % adverse events in placebo group were reported. Amyloid-related imaging abnormalities (ARIA) corresponding to microhemorrhages were observed in 3 patients treated with CAD106. No vasogenic edemas were observed [54]. Novartis and the Banner Health Institute have begun phase II/III trials testing this novel vaccine with a BACE I inhibitor in pre-symptomatic, cognitively healthy adults, who have two copies of the ApoE4 gene, making them more at risk of developing AD, in a trial known as the Alzheimer’s Prevention Initiative APOE4 trial, expected to reach completion in 2023 (www.alzforum.org).

Also, currently in clinical phase I/II trials is AC Immune’s ACI-24 liposome-based vaccine using Aβ1-15, which aims to produce β-sheet conformation-specific antibodies (www.acimmune.com). AC Immune, together with the LuMind Research Down Syndrome Foundation, have brought this vaccine into phase I trials in 24 adults with Trisomy 21-positive Down Syndrome, the results of which are expected in 2019 (https://clinicaltrials.gov). In addition, UB-311 (United Biomedical), targeting Aβ1-14, has shown safety and efficacy in phase I clinical trials with a phase II study currently recruiting patients with mild AD (https://clinicaltrials.gov) and V950 from Merck, an N-terminal Aβ fragment conjugated to an aluminum-containing adjuvant with or without ISCOMATRIX, has completed a phase I clinical trial.

Finally, an active vaccine developed by AFFiRiS AG, Austria stimulating an immune response against Aβ by short peptides obtained by molecular mimicry (“mimotopes”) [55] has completed phase II clinical trials. Their front-runner AD02 stimulates an immune response directed towards the N-terminus of unmodified Aβ species (https://clinicaltrials.gov). At a press briefing in June 2014, AFFiRiS reported first results on the efficacy of AD02 showing that AD02 did not reach primary or secondary outcome measures (www.alzforum.org). According to AFFiRiS (12th International Conference on Alzheimer’s and Parkinson’s Diseases and Related Neurologic Disorders, AD/PD 2015, Nice), placebo-treated patients showed less cognitive decline accompanied by less hippocampal shrinkage than AD02-treated patient. Whether the ingredients of the placebo formulation are indeed responsible for the observed effects remains to be determined. Nevertheless, the same formulation was also used for application of AD02, confusing the potential causes of this trial failure (www.alzforum.org).

### Latest developments in passive Aβ immunotherapy

Following the termination of the AN1792 trial, significant effort has gone into the development and testing of monoclonal antibodies for passive Aβ immunization. The first antibody in clinical trials, bapineuzumab (Janssen and Pfizer), is the humanized version of 3D6, an IgG1 mouse monoclonal specific for an N-terminal Aβ epitope (Aβ1-5) and it only recognizes Aβ peptides with an aspartic acid residue at the N-terminus [56]. Although phase II clinical trials in mild-to-moderate AD did not initially present clear clinical benefits, *post-hoc* analysis revealed that a subgroup of ApoE4 non-carriers showed some evidence of clinical improvement. Furthermore, 12/124 (9.7 %) of bapineuzumab-treated patients experienced transient and reversible edema [also known as ARIA-E], which was more frequent in ApoE4 carriers with one copy of the allele (7.1 %) and two copies of the allele (33.3 %) compared to non-carriers (4.3 %). In addition, 11/12 edema cases occurred at a dose >1 mg/kg [57]. Whether the observed edema is vasogenic or due to other mechanisms such as microglial activation requires further studies. Target engagement detected by ^11^C-PiB PET imaging showed evidence for a stabilization of Aβ burden in bapineuzumab-treated subjects compared to an increase in the placebo-treated individuals [58]. These findings and modest clinical benefits warranted continued evaluation in phase III trials. Unfortunately, two large phase III clinical trials reported no significant clinical benefits, which led to the termination of all phase III bapineuzumab clinical trials. Recent biochemical analyses of the brains from 3 patients treated with bapineuzumab showed a trend for reduction in Aβ42 accompanied by an increase in Aβ40 leading to an overall reduction in the Aβ42/40 ratio. These findings highlight a dynamic homeostatic balance of Aβ, obviously altered by bapineuzumab treatment [59]. In addition, AAB-003, a derivative of Bapineuzumab with altered effector function was tested in Phase I studies with results pending (www.alzforum.org).

Eli Lilly’s solanezumab is the humanized version of m266, an IgG1 monoclonal antibody that recognizes an epitope within Aβ16-24. It is purported to preferentially recognize soluble Aβ species and not fibrillar Aβ and has been shown to increase both plasma and CSF Aβ [60]. Solanezumab was tested in phase III clinical trials and failed to show significant improvement in primary outcomes in the phase 3 trials, EXPEDITION 1 and EXPEDITION 2 [61]. Secondary analysis in pooled subjects from both trials with mild AD showed less cognitive and functional decline when treated with solanezumab compared to placebo. In contrast, patients with moderate AD did not benefit from solanezumab treatment [62]. Unlike bapineuzumab, solanezumab has not been associated with ARIA-E or microhemorrhages [60] and is well tolerated at doses up to 400 mg administered weekly. An additional phase III study testing solanezumab at 400 mg every 4 weeks for 76 weeks in mild AD patients (EXPEDITION III), is currently ongoing and expected to reach completion in 2018 (https://clinicaltrials.gov).

Most recently, aducanumab, a co-development of Neurimmune (Switzerland) and Biogen (USA) has gained much attention. Aducanumab (BIIB037) results from reverse translational medicine and is not a humanized mouse monoclonal antibody. Instead, it is a fully human IgG1 monoclonal antibody isolated from healthy aged donors representing cognitively healthy aging (www.alzforum.org). As presented at the 11^th^ International Conference on Alzheimer’s and Parkinson’s Diseases and Related Neurological Disorders held in 2013 in Florence, Italy, aducanumab binds fibrillar amyloid and is able to reduce plaque burden in AD mice. Aducanumab was tested in prodromal or mild AD and showed a dose-dependent reduction of Aβ-PET signal almost to threshold levels and slowing of cognitive decline at the highest dose (10 mg/kg) as presented at AD/PD 2015 in Nice and the Alzheimer’s Association International Conference (AAIC) 2015 in Washington DC. The observed effect on cognitive decline was dose-dependent. ARIA-E was reported to increase with dose and ApoE4 carriage with 55 % incidence in homozygous ApoE4 carriers (www.alzforum.org). Nevertheless, this represents a breakthrough in AD immunotherapy and the data encouraged the development team to take aducanumab directly to phase III clinical studies.

In addition to the clinical development of monoclonal Aβ antibodies, consortia were established to study the effect of preventive passive immunization in different study populations. In this regard, the “Dominantly Inherited Alzheimer’s Network” (DIAN) is testing treatments in patients with FAD mutations that puts them at a greater risk for developing AD than the general population. The monoclonal antibodies solanezumab (Lilly) and gantenerumab (Roche) were chosen as the two investigational antibodies. Gantenerumab, an IgG1 antibody, recognizes two epitopes in Aβ. One lies within the N-terminus and another within the mid-region of Aβ. Gantenerumab was further shown to preferentially bind fibrillar Aβ [63]. This study is scheduled for completion at the end of 2016.

Furthermore, the “Alzheimer’s Prevention Initiative” (API) is currently enrolling 300 Columbian individuals harboring an autosomal-dominant E280A PS1 mutation, which has been associated with early-onset AD [64, 65]. Because of its IgG4 backbone designed to engage microglial-mediated phagocytosis of Aβ but not to induce a pro-inflammatory response, crenezumab (Genentech) has been selected. Crenezumab is the humanized version of antibody mMABT generated by immunizing mice with a liposome-coupled N-terminal fragment of Aβ (Aβ 1-15) termed AC-01 by the Switzerland-based company AC Immune [66, 67]. Crenezumab was described to bind different aggregated forms of Aβ, including oligomers and fibrils and to possess a low affinity for Aβ monomers [67].

Finally, unlike the two aforementioned initiatives in FAD subjects, the “Anti-Amyloid Treatment for Asymptomatic Alzheimer’s Disease” (A4) is enrolling 1000 subjects between 65–85 years of age w/o dominantly inherited early onset AD mutations, who exhibit amyloid in brain by PET imaging but are otherwise cognitively normal. This study targets people that are at risk for developing sporadic AD and represents the majority of the AD population. Because of its known safety profile and evidence of modest cognitive benefit in mild AD patients, solanezumab was chosen for the A4 trial (www.alzforum.org).

### Rationale for immunization against pGlu-3 Aβ

Most of the Aβ immunotherapy clinical trials have not reached primary endpoints thus far, although some positive effects were observed in *post-hoc* analyses, e.g. in the Solanezumab Expedition Trials. Aside from known problems with selecting the right study population and the optimal cognitive stage of a patient for starting treatment, current immunotherapy strategies may not be targeting Aβ peptides with high toxic potential such as pGlu-3 Aβ [34]. In this regard, pGlu-3 Aβ appears to be an attractive novel target for Aβ vaccination approaches as Aβ1-40 and Aβ1-42 are products of normal APP turnover [68]. In addition, Aβ is produced at similar levels by young and aged individuals [69]. Its production rate in healthy volunteers and AD patients does not significantly differ, e.g. Aβ42 is generated with a rate of 6.7 %/h in non-AD and 6.6 %/h in AD patients as measured by the increase of ^13^C_6_-leucine labeled Aβ in CSF over time [70]. Therefore, it is reasonable to consider Aβ1-40 and Aβ1-42 as physiologic peptides, although their exact biologic role is still not well understood. Recent investigations have shown an anti-microbial activity of Aβ as well as an importance for neuronal physiology [71, 72]. Thus, active vaccination against full-length Aβ peptides early in life might eliminate an important molecule of innate immunity and neuronal physiology with unknown outcome.

In contrast to the physiologic generation of Aβ1-40 and Aβ1-42 peptides, pGlu-3 Aβ, the result of a significant side-reaction of QC, has a dramatic influence on the amyloidogeneity and toxicity of the Aβ molecule. Therefore, pGlu-3 Aβ represents a solely non-physiologic neoepitope (Fig. 1) that could be targeted by different immunotherapy approaches, sparing the presumably physiologic Aβ molecules 1–40 and 1–42.

### Active vaccination against pGlu-3 Aβ

The development of second-generation active vaccines was initiated after failure of the AN1792 trial, however clinical testing has not caught up with extent of passive immunization clinical studies performed by peripheral application of humanized anti-Aβ antibodies. Thus, the reports on active vaccination against pGlu-3 Aβ are scarce and publicly reported clinical development is currently limited to a single biopharmaceutical company, AFFiRiS AG (Austria). Here, the active pGlu-3 Aβ vaccine (“mimotope”) was obtained by molecular mimicry resulting in an unrelated peptide stimulating antibody production against pGlu-3 Aβ (Table 1) [55]. This strategy circumvents a T-cell response suspected of causing clinical side effects in the AN1792 trial. Although no peer-reviewed publications are available for the preclinical testing of anti-pGlu-3 Aβ mimotopes, according to a published patent, the company claims a 38 % reduction in the area occupied by amyloid plaques in Tg2576 mice treated (s.c.) over 6 months using an anti-pGlu-3 Aβ mimotope with aluminum hydroxide (ALUM) as an adjuvant. The mice were sacrificed at 13 months of age and plaque area was determined by an Aβ40/42-specific antibody (Mandler M. *et al*. (2011) US Patent Application No: 20110097351). A Phase 1 clinical trial in mild AD patients of the active vaccine including the pGlu-3 Aβ mimotope and alum adjuvant, AD03, was completed in 2011 with promising results, followed by a Phase 1b study that was terminated in 2013 due to organizational reasons (https://clinicaltrials.gov). The outcome of the study has not been disclosed thus far.

**Table 1:** Summary of preclinical results obtained by active and passive vaccination

Ag/Ab	AD Model	Treatment	Effect	Ref.
Active vaccination				
pGlu-3 mimotope	Tg2576	Preventive	Reduced plaques	USPTO No.: 20110097351
pGlu-3-9-KLH	J20	Preventive	Reduced plaques	[73]
pGlu-3 Aβ42	Rabbit (WT)	-	-	[74]
Passive vaccination				
9D5	5XFAD	Preventive	Reduced plaques and Aβ peptides	[76]
07/1	APP/PS1ΔE9	Preventive & Therapeutic	Reduced plaques; no change in Aβ peptides	[77–80]
mE8	PDAPP	Therapeutic	No change in plaques; reduced Aβ peptides	[83]

*Ag* Antigen, *Ab* Antibody, *Ref*. Reference, *USPTO* US Patent and Trademark Office

At the Society for Neuroscience meeting held in San Diego in 2010, our own group reported the active vaccination of 6-month-old J20 mice with the N-terminal Aβ fragment pGlu-3–9 coupled to KLH in comparison to an aged 3:1 mixture of Aβ1-40 and Aβ1-42, each with CFA/IFA adjuvant (Table 1). J20 mice first start to develop pGlu-3 Aβ containing plaques at 6 months of age, albeit in low quantities [20]. Mice were immunized (i.p.) on days 0, 14 and 28 followed by monthly injections for 8 months and generated high titers of antibodies specific for the N-terminus of their respective antigens. Both vaccines resulted in a significant reduction of amyloid plaques, indicating that pGlu-3 Aβ-targeted active immunization prevented deposition of both pGlu-3 Aβ and general Aβ with heterogeneous N-termini [73]. This underlines the seeding capacity of pGlu-3 Aβ and is in line with observations made by application of QC-specific inhibitors preventing pGlu-3 Aβ formation [21].

Finally, active vaccination of rabbits using pGlu-3 Aβ42 resulted in antibodies exclusively detecting this Aβ isoform (Table 1). No cross-reactivity was observed to Aβ1-42 or pGlu-11 Aβ [74]. The immuno-dominant region of pGlu-3 Aβ was identified within the N-terminus with no cross-reactivity to unmodified peptides starting with N-terminal glutamate [74]. This is in contrast to active vaccination using Aβ1-40 or Aβ1-42, where the immuno-dominant epitope was represented by the amino acid sequence EFRH in the N-terminal region of full-length Aβ [75]. These data are corroborated by our own observations wherein immunization with pGlu-3–9-KLH dominantly produced pGlu-3 Aβ specific antibodies whereas immunization with Aβ1-40/42 produced N-terminal (Aβ 1-15) specific antibodies. Aside from AFFiRiS’s AD03 mimotope vaccine, as far as we are aware, no other active anti-pGlu-3 Aβ vaccine has progressed to clinical development to date.

### Passive immunization against pGlu-3 Aβ

Passive anti-pGlu-3 Aβ immunotherapy has been more frequently reported in the literature than active vaccination although the total number of publications is still very low. The first report on passive pGlu-3 Aβ immunotherapy was published in 2010 [76]. Thomas Bayer at the University of Goettingen (Germany) generated a novel IgG2b monoclonal antibody (9D5) preferentially targeting oligomeric aggregates of pGlu-3 Aβ (Table 1) and demonstrated that passive immunization (i.p.) of 250 μg 9D5 in 4.5-month-old 5XFAD mice over 6 weeks reduced hippocampal and cortical total Aβ, Aβ40, Aβ42 and pGlu-3 Aβ plaque load and intracellular pGlu-3 Aβ oligomers [76]. The study further showed a significant reduction of pGlu-3 Aβ in TBS and SDS fraction of brain lysates detected by ELISA. 5XFAD mice display general Aβ deposition as early as 1.5 months of age in cortical layer V and the subiculum. Detection of pGlu-3 Aβ starts at 4 months of age in focal plaques throughout cortical layer V and the subiculum, followed by detection in the granule cell layer of the dentate gyrus at 6 months of age, and pGlu-3 Aβ positive focal deposits throughout the entire brain at 9–12 months of age [23]. Therefore, the study published by Bayer and colleagues started treatment around the onset of first pGlu-3 Aβ deposition but after the onset of general Aβ deposition and therefore, can be considered a prevention trial.

Two pilot studies published in 2012 by our own lab further strengthened the feasibility of passive pGlu-3 Aβ immunization (Table 1) [77]. Here, APP/PS1ΔE9 mice underwent preventive and therapeutic passive immunization using a novel pGlu-3 Aβ specific antibody (07/1, IgG1) developed by Probiodrug AG (Germany). In APP/PS1ΔE9 mice, general Aβ deposition starts at approximately 5 months of age in the hippocampus with a subset of plaques also positive for pGlu-3 Aβ. At 14 months of age, pGlu-3 Aβ is found in diffuse and focal deposits in cortex, hippocampus and cerebellum. By 24 months of age, these mice display abundant pGlu-3 Aβ immunoreactivity of plaques within hippocampus and a subset of plaques in the neocortex [23]. The pilot prevention trial was started in mice at 5–6 months of age, at the onset of plaque deposition. Passive immunization (i.p.) with 200 μg of 07/1 over 32 weeks resulted in a significant reduction in the deposition of general Aβ and pGlu-3 Aβ and Thioflavin S-positive fibrillar Aβ in the hippocampus as detected by immunohistochemistry (IHC). In addition, significant reductions in general Aβ and pGlu-3 Aβ deposition were observed in the cerebellum, an area prone to plaque deposition in this model [23]. In contrast to the study from Wirths et al. [76], we observed no significant reduction of pGlu-3–42 or total Aβ42 measured by ELISA in guanidine HCl-extracted hemibrains. The reduced plaque load observed in the pilot passive immunization prevention trial was further validated in a larger study by our lab in which cognitive performance of 07/1-vaccinated APP/PS1ΔE9 mice was normalized to wildtype levels following treatment [78].

The pilot therapeutic trial in plaque-rich 23-month-old mice using weekly 07/1 antibody injections (200 μg) for 7 weeks demonstrated a plaque-lowering effect [77]. A more recent larger study from our lab has confirmed and extended these results to include behavioral analyses [79–81]. Interestingly, we demonstrated in our pilot studies a similar strong reduction of amyloid plaque load and fibrillar Aβ in aged APP/PS1ΔE9 mice immunized for only 7 weeks compared to that in the prevention study over 32 weeks in young mice. This was even more pronounced in the cerebellum, an area of late plaque deposition in this model, suggesting that the antibody may be most efficacious in preventing and/or removing newly deposited plaques. This result is consistent with the change in Aβ40/42 ratio observed in patients immunized with bapineuzumab [59], and suggests that passive immunization using a pGlu-3 Aβ antibodies is able to change parameters of pathology long after the onset of amyloid deposition. Importantly, unlike previous reports that passive immunization with N-terminal Aβ antibodies increased the incidence of microhemorrhages in aged APP23 mice [82], we have observed no increase in microhemorrhages in 07/1 pGlu-3 Aβ-immunized APP/PS1ΔE9 mice to date.

A recent publication from researchers at Eli Lilly and Company described the effects of a novel pGlu-3 Aβ specific IgG2a antibody (mE8) on Aβ levels and plaque pathology in aged PDAPP mice (Table 1) [83]. The results were compared to the 3D6 IgG1 antibody, which represents the murine precursor of bapineuzumab. Aged PDAPP mice (23–24 months) were treated for 3 months with 12.5 mg/kg mE8 and 3D6. At the start of treatment the mice showed a similar proportion of pGlu-3-modified Aβ42 as observed in human AD brain tissue. The applied dose translated into approximately 500 μg antibody/mouse, which is 2.5X the amount used in our own pilot study. DeMattos et al. were able to show a significant reduction of guanidine-HCl extractable Aβ1-42 in hippocampus. The effect was dependent on the effector function of the respective IgG molecule, with mE8-IgG2a being more effective than mE8-IgG1. This was in line with the abilities of different mE8-IgG subclasses to stimulate *ex vivo* phagocytosis of Aβ by primary murine microglia cells. The effect on Aβ1-42 was less pronounced in cortex, where only treatment with mE8-IgG2a reached statistical significance. In addition, quantification of the total plaque area by immunohistochemical staining failed to show differences among the treatment groups although the authors reported a reduction of existing “plaque load” by mE8 treatment using biochemical measurements (ELISA) [77, 83]. This is in contrast to our published pilot study, where significant reductions in both general and pGlu-3 Aβ labeled plaques were observed [77]. More recently, Lilly reported at the Alzheimer’s Association International Conference held in Copenhagen in 2014 that co-treatment of mice with their pGlu-Aβ specific antibody mE8 together with a BACE I-inhibitor cleared both compacted and diffuse plaques in aged PDAPP mice, suggesting that the BACE I inhibitor led to a reduction of diffuse plaques while the pGlu-Aβ antibody to a reduction of compacted plaques. The combination cleared both types of amyloid deposits suggesting a synergistic effect of such combination therapy (www.alzforum.org). The clinical candidate is termed LY3002813 and an ongoing clinical phase 1 trial is aiming at determining safety and tolerability in up to 100 healthy participants as well as in subjects with MCI and mild to moderate AD patients (https://clinicaltrials.gov).

## Conclusions

AD brains contain considerable amounts of pGlu-3 Aβ although absolute values differ between the methods used for quantification. Results obtained by ELISA showed that pGlu-3 Aβ42 accounts for less than 1 % of total Aβ42 [21]. Other studies using mass spectrometry calculated quantities of up to 27 % of all Aβ peptides to start with an N-terminal pGlu-residue in temporal cortex [22]. In cotton wool plaques deposited in patients carrying the PS1 V261I mutation, N-truncated and pGlu-modified Aβ peptides represent the major species [6]. Therefore it is unclear, why pGlu-3 Aβ peptides did not gain much attention. One explanation might be that the pGlu-modification was frequently considered as secondary reaction occurring later in disease progression [84].

Although the absolute amount of deposited pGlu-3 Aβ varies between different reports, preclinical studies aiming at reducing pGlu-3 Aβ peptides in brains of AD-like mouse models have shown remarkable success. The ability to reduce general Aβ deposition by pGlu-3 Aβ-specific immunotherapy is comparable to the efficacy seen with immunization targeting full-length Aβ [83].

It has been suggested that pGlu-3 Aβ is a seeding species for general Aβ deposition. Furthermore, pGlu-3 Aβ appears to cause template-induced misfolding of other Aβ molecules already apparent in very low quantities [34]. Active and passive immunization approaches as well as pharmacologic inhibition of pGlu-3 Aβ formation by QC inhibitors in mouse models with considerable lower pGlu-3 Aβ deposition compared to humans [20] now substantiates these biophysical and cell biological studies [21, 33, 34]. Due to its nature as a by-product of a catalytic side-reaction of QC, pGlu-3 Aβ further represents a true Aβ neoepitope that only correlates with deleterious effects on brain physiology.

Despite these encouraging results, immunotherapy against pGlu-3 Aβ faces similar challenges as approaches directed against other Aβ isoforms. These include: (I) Passively administered pGlu-3 Aβ antibodies need to efficiently cross the blood brain barrier to reach target molecules in the brain. (II) Treatment needs to be initiated as early as possible since active and passive immunization in patients already diagnosed with AD has been incapable of stopping disease progression thus far. (III) Active immunization against pGlu-3 Aβ in AD patients would also face the problem of immunosenescence leading to a certain percentage of non-responders. (IV) The application of humanized monoclonal antibodies may induce an immune response against the treatment antibodies reducing their efficacy. (V) pGlu-3 Aβ appears to be specifically present in brain parenchyma and absent in detectable amounts in body fluids such as CSF or plasma [83]. Given its neurotoxic nature, it is unclear what adverse effects are to be expected when this exceptionally toxic variant is mobilized from deposits of PiB-positive individuals by Aβ immunotherapy. On the other hand, the absence of detectable levels of pGlu-3 Aβ in plasma may reduce peripheral saturation thereby allowing more antibodies for delivery to the brain parenchyma.

In the future, the current clinical trials (DIAN, API, A4) that aim to prevent AD symptoms in people at risk, will help to further illuminate the way for AD immunotherapy, including immunization targeting pGlu-3 Aβ.

## Abbreviations

A4, Anti-Amyloid Treatment for Asymptomatic Alzheimer’s Disease; AAIC, Alzheimer’s Association International Conference; AD, Alzheimer’s disease; AD/PD, International Conference on Alzheimer’s Disease & Parkinsons’s Disease; API, Alzheimer’s Prevention Initiative; ApoE4, Apolipoprotein E4 allele; APP, amyloid precursor protein; ARIA, amyloid-related imaging abnormalities; Aβ, beta-amyloid peptide; BACE, beta-site APP cleaving enzyme; CFA, Complete Freund adjuvant; CSF, cerebrospinal fluid; DIAN, Dominantly Inherited Alzheimer’s Network; FAD, familial form of Alzheimer’s disease; IFA, incomplete Freund adjuvant; IgG, immunoglobulin G; KLH, keyhole limpet hemocyanin; MCI, mild cognitive impairment; PET, Positron emission tomography; pGlu, pyroglutamate, pyroglutamyl-; PiB, Pittsburgh compound B; PS-1, Presenilin 1; QC, Glutaminyl Cyclase.
